# Microwave excitation of spin wave beams in thin ferromagnetic films

**DOI:** 10.1038/srep22367

**Published:** 2016-03-14

**Authors:** P. Gruszecki, M. Kasprzak, A. E. Serebryannikov, M. Krawczyk, W. Śmigaj

**Affiliations:** 1 Faculty of Physics, Adam Mickiewicz University in Poznań, Umultowska 85, 61-614 Poznań, Poland; 2 Simpleware Ltd., Bradninch Hall, Castle Street, Exeter, EX4 3PL, UK

## Abstract

An inherent element of research and applications in photonics is a beam of light. In magnonics, which is the magnetic counterpart of photonics, where spin waves are used instead of electromagnetic waves to transmit and process information, the lack of a beam source limits exploration. Here, we present an approach enabling generation of narrow spin wave beams in thin homogeneous nanosized ferromagnetic films by microwave current. We show that the desired beam-type behavior can be achieved with the aid of a properly designed coplanar waveguide transducer generating a nonuniform microwave magnetic field. We test this idea using micromagnetic simulations, confirming numerically that the resulting spin wave beams propagate over distances of several micrometers. The proposed approach requires neither inhomogeneity of the ferromagnetic film nor nonuniformity of the biasing magnetic field. It can be generalized to different magnetization configurations and yield multiple spin wave beams of different width at the same frequency.

Magnonics is an emerging field of research and technology whose area of interest is spin wave (SW) dynamics^1^. In many aspects, it is closely related to photonics, which deals with electromagnetic waves, and phononics, which is concerned with elastic waves. Generation^2^,^3^, transmission^4^,^5^, signal processing^6^, amplification^7^, and detection^8^ of SWs are presently major subjects of study in magnonics. Basic components performing these operations in nano- and mesoscale have recently been demonstrated. However, their functionality and performance require significant improvement to make magnonic elements competitive (in terms of energy efficiency, throughput, etc.) with other kinds of integrated devices: electronic, photonic, and acoustic^9^,^10^. The theoretical underpinnings of magnonics also constitute a rich and still not fully explored research area. SW caloritronics^11^, magnonics-spintronics^12^,^13^, magnonic crystals^14^, magnonic metamaterials^15^, and Bose-Einstein condensates of magnons^16^ are some of the topics whose investigations have been started in recent years.

The crucial milestone in modern photonics from both the scientific and the technological perspective was the development of efficient sources of coherent light beams: lasers. In magnonics, an efficient source of coherent SW beams is not yet available, in spite of many attempts of its development. The caustic effect^17^,^18^, nonlinear self-focusing of SWs^19^,^20^,^21^, and nonuniform internal magnetic field^22^,^23^ are among the beam excitation mechanisms investigated to date. The first approach is limited to low frequencies, i.e., to magnetostatic SWs, which have a caustic dispersion relation. SW can propagate only at specific angles with respect to the direction of the magnetization vector, which needs to be in the plane of the film. The second approach requires excitation of SWs with high amplitude; moreover, the beam spreads quickly after passing the focal point, which strongly limits its usefulness. In the third approach, SWs propagate in channels of the static magnetic field with decreased magnitude; generation of such an nonuniform magnetic field is technologically complex.

On the other hand, electromagnetic wave transducers—microstripe and coplanar waveguides (CPWs)—are extensively used in studies of SWs in thin ferromagnetic films^24^,^25^ as sources of plane-wave-like SWs. These transducers enable direct coupling of the microwaves and SWs, a property that can be crucial for microwave applications of magnonics^26^. In this paper, we propose and numerically validate with micromagnetic simulations (MSs) a method of excitation of narrow SW beams in homogeneous ferromagnetic thin films using the microwave-frequency (mf) magnetic field generated by CPW transducers with a suitable geometry. We exploit the fact that efficient excitation of an SW can occur only when the Fourier transform of the mf magnetic field has a large magnitude at the wave vector equal to the SW wave vector at the excitation frequency. To ensure that this condition holds only locally, we vary the transducer’s profile along the mf current flow direction. This variation affects the spatial distribution of the magnetic field induced by the current and, in particular, its Fourier spectrum along the expected direction of SW propagation. In its most basic form, this method enables excitation of a single, well localized SW beam propagating perpendicularly to the waveguide axis. By adjusting the transducer’s geometry, this approach can be generalized, so that multiple SW beams of identical or different width may simultaneously appear in different regions of the ferromagnetic film. Other possible scenarios and applications will be also discussed in this paper.

## Results

### Basic principles

Throughout this paper, we consider propagation of SWs in a ferromagnetic film of thickness 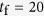 nm made of yttrium-iron garnet (YIG). YIG is a promising dielectric material with the lowest SW damping ever recorded. Recently, the technology of ultra-thin YIG film deposition has been developed, holding promise for integrated magnonics^27^,^28^. Unless stated otherwise, we assume the film to be saturated normal to its plane, i.e., along the *z* axis, by an external magnetic field 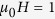 T, where *μ*_0_ is the permeability of vacuum. We take the YIG magnetization saturation to be 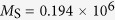 A/m, exchange constant 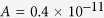 J/m, and gyromagnetic ratio 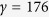 rad GHz/T^29^.

**Figure 1:**
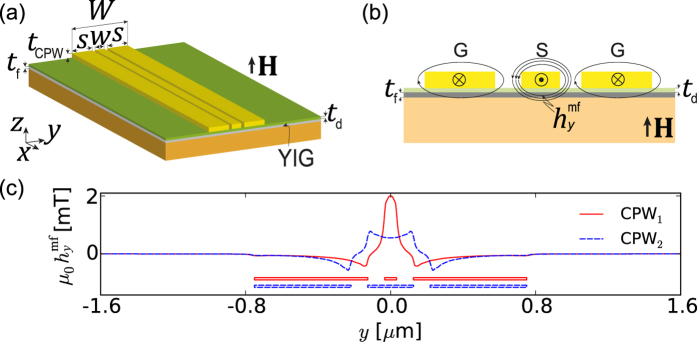
Coplanar waveguide geometry. (**a**) Perspective view and (**b**) cross-section of a CPW consisting of a signal line (denoted by S) and two ground lines (denoted by G). A ferromagnetic yttrium-iron-garnet (YIG) film of thickness 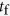 is separated from the CPW by a nonmagnetic, dielectric layer of thickness 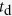. The YIG is saturated normal to the film plane by an external magnetic field *H*. The mf current transmitted along the *x* axis generates a magnetic field 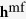, which exerts a torque on the magnetization in YIG. (**c**) The *y* component of the mf magnetic field along the *y* axis, 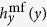, excited by 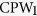 (red line) and 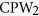 (blue line), obtained from CST simulations. The horizontal bars at the bottom of this plot show the widths of 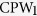 (upper) and 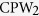 (lower) ground and signal lines.

Let us briefly revisit SW excitation by mf current flowing through the uniform CPW transducer shown in Fig. 1a and b. The CPW consists of a signal line (S) of width *w* separated by gaps of width *s* from two identical ground lines (G). The signal and ground lines are deposited on a nonmagnetic, dielectric layer of thickness 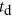 covering the ferromagnetic film. We assume that the ferromagnetic film is deposited on the same nonmagnetic dielectric. A microwave signal in the CPW generates a mf magnetic field 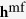, which oscillates in the 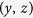 plane, i.e., perpendicular to the CPW axis, see Fig. 1b. In the plane of the ferromagnetic film, only the *y* component of this field exerts a non-zero torque on the magnetization, which is parallel to the *z* axis. This torque induces coherent magnetization precession around the equilibrium direction and causes generation of SWs propagating along the *y* axis^25^.

Figure 1c shows the profile of the *y* component of the mf magnetic field 7.5 nm below the ground and signal lines of two CPWs, CPW_1_ and CPW_2_, made of Cu with conductivity 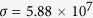 S/m^30^ and thickness 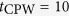 nm, operating at frequency 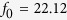 GHz. The dielectric layer has thickness 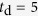 nm and relative permittivity 14.7 (close to the permittivity of gadolinium-gallium garnet, a substrate used in fabrication of YIG). The width of the signal line in 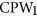 is 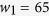 nm and in 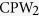, 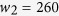 nm. The total width of the transducers and the width of the gap between the signal and ground lines are the same in both waveguides: 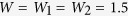 *μ*m and 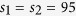 nm. The magnetic field distributions are obtained from CST simulations (see Methods).

Next, let us discuss the basic principle of selective (local) wave vector matching. Following ref. 8, we assume that the efficiency of monochromatic SW excitation by a CPW depends, in particular, on the quality of the match between the wave number of the SW at the excitation frequency 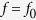, 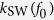, and the wave number 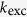 corresponding to the maximum of the Fourier transform (FT) of the microwave magnetic field 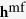 oscillating at the same frequency. Thus, the most efficient excitation of SWs is expected when


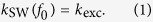


On the other hand, if the magnitude of the FT at an SW wave number 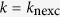 is very small, the matching condition (1) is not fulfilled and no efficient SW excitation should arise at the selected frequency. These features make it possible to engineer the wavefronts of excited SWs, and in particular to excite SW beams with high efficiency, as will be demonstrated below.

**Figure 2:**
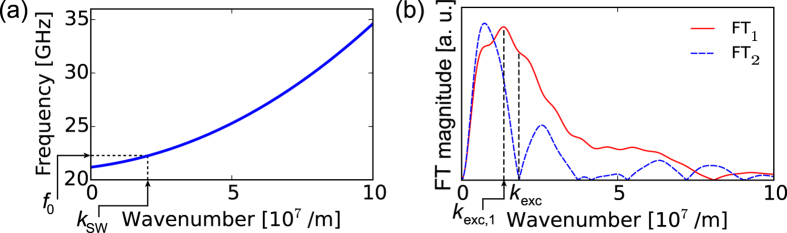
Correspondence between spin wave dispersion relation and the spectral distribution of the exciting mf magnetic field. (**a**) The dispersion relation of spin waves (SWs) in a 20-nm thick YIG film with out-of-plane magnetization, placed in an external magnetic field of 1 T. (**b**) Magnitude of the Fourier transforms FT_1_ and FT_2_ of the mf magnetic fields 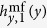 and 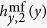 emitted by two CPWs, CPW_1_ and CPW_2_, at frequency *f*_0_ (see Fig. 1). The maximum of 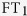 is located at 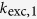. At 
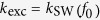
, i.e., the wave number equal to the wave number of SW at frequency 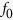 (see plot **a**), the value of FT_1_ is still close to the maximal one, while *FT*_2_ has a minimum.

Figure 2a shows the dispersion relation of SWs in a YIG film saturated along the normal to its plane, which is calculated with the analytical formula^29^


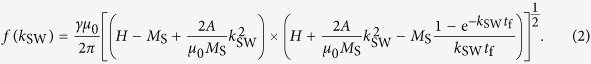


In line with equation (2), the dispersion is independent from the direction of the wave vector and has a predominantly parabolic behavior in the considered wave number range.

As discussed above, SWs are excited mainly by the mf magnetic field component parallel to the expected direction of their propagation, further denoted by *y*. Thus, we focus now on the behavior of 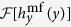, the FT of 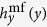. Figure 2b shows the Fourier transforms 
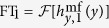
 and 
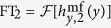
 of the magnetic fields generated by the CPW_1_ and CPW_2_ transducers, respectively. At frequency 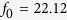 GHz equation (1) holds approximately only for FT_1_ [near 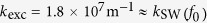, see Fig. 2], while the magnitude of FT_2_ is minimal. Therefore, one may expect that at *f* = *f*_0_ SWs can be effectively excited only with the field 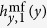.

**Figure 3:**
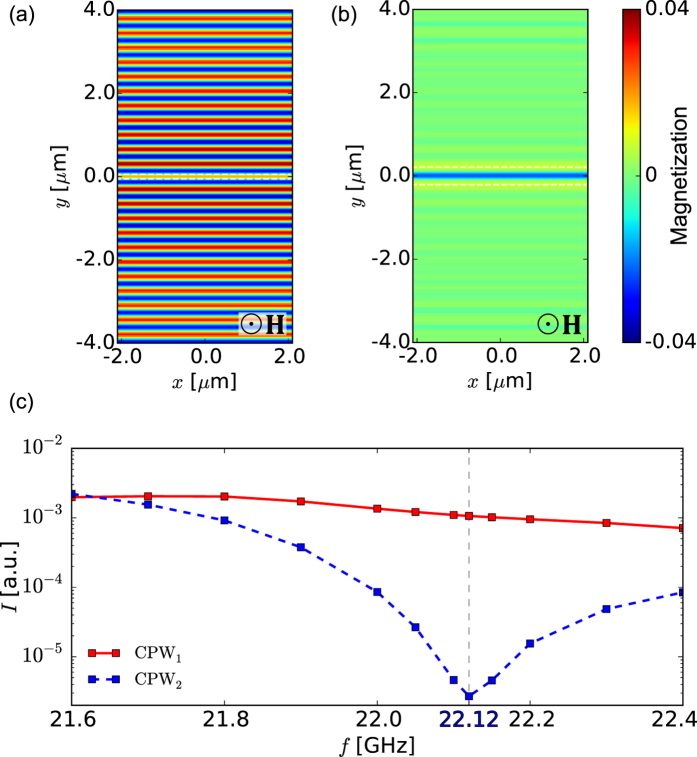
Efficiency of SWs excitation using different CPW antennas. (**a**), (**b**) Dynamic component of the magnetization vector (perpendicular to the film plane) of the SWs induced by the mf magnetic field from (a) CPW1 and (b) CPW2 at 22.12 GHz, obtained from MSs. The horizontal white dashed lines correspond to the signal line width. (**c**) Dependence of the efficiency of SW excitation (in the logarithmic scale) in the YIG film by CPW1 (red solid line) and CPW2 (blue dashed line) on the frequency of the mf current. Intensity (*I*) represents squared amplitude of the dynamical magnetization averaged over four periods calculated at 2 *μ*m distance from the excitation. The efficiency of the SWs excitation by CPW2 reaches a minimum at *f* = 22.12 GHz.

Frequency *f*_0_ is achievable by standard microwave generators, and at this frequency the dispersion relation of SWs in YIG thin film is almost isotropic also when the magnetization saturation is in-plane of the film. This makes the results of this study more general. However, at small enough wave vectors the strong anisotropy of the dispersion relation has to be taken into account. Indeed, Fig. 3a,b show the SWs induced by 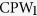 and 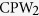 at 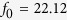 GHz, i.e., when 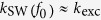 for the former and 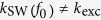 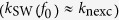 for the latter. Note that in CPW_2_ 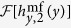 is close to 0 at 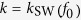, see Fig. 1b. As expected, the propagating SWs are excited efficiently only in CPW_1_, for which condition (1) is satisfied. Figure 3c demonstrates that the intensity of SWs excited by CPW_1_ remains two orders of magnitude over that of CPW_2_ within a 50 MHz-wide frequency range centered around 22.12 GHz.

If the transducer is designed so that it produces the magnetic field distribution 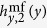 over most of its length and 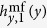 only along a short section, an SW will be excited by mf current oscillating at frequency 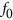 only in that short section. Provided that this section is at least a few times longer than the SW wavelength 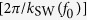, this should give rise to a well-collimated SW beam. Beam excitation in a wide frequency band will be possible if the dip in FT_2_ near 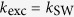 (see Fig. 3c) is sufficiently broad and the slope of the SW dispersion relation near 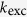 (Fig. 2a) is sufficiently steep. These parameters can be optimized by modifying the CPW geometry and the ferromagnetic film or magnetic field, respectively.

**Figure 4:**
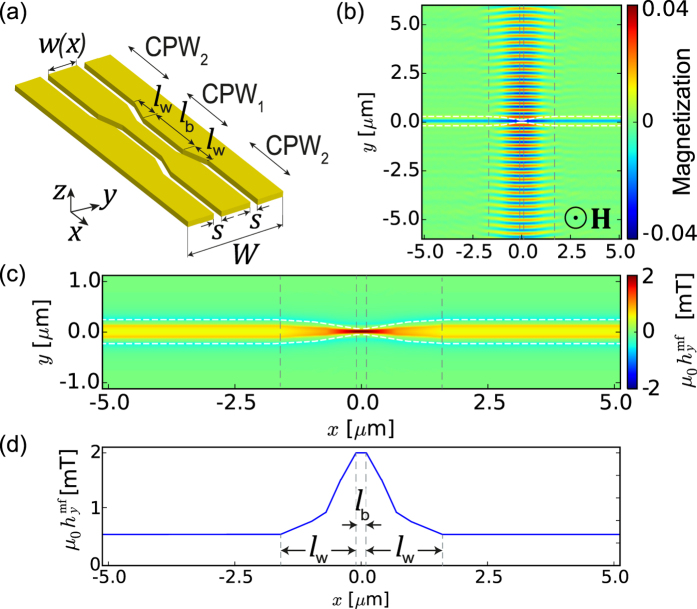
Spin wave beam excitation using the nonuniform CPW. (**a**) Geometry of the nonuniform CPW proposed to excite an SW beam. (**b**) Dynamic component of the magnetization vector of the SW beam excited in a thin YIG film by the mf magnetic field (shown in plot (**c**)) induced by the nonuniform CPW (results of MSs). (**c**) Distribution of the mf magnetic field 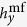 induced by the CPW on the top surface of the YIG film. The shape of the signal line is marked with dashed white lines. (**d**) Profile of 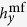 along the axis of the signal line, 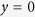.

Following the above prescription, to restrict SW generation to a short section of the transducer and hence to obtain a clearly defined beam, we vary the CPW geometry along the *x* axis. The signal line width is changed from 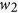 at 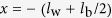 to 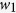 at 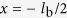 and back from 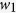 at 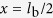 to 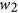 at 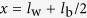, as shown in Fig. 4a. The gap width 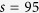 nm and the total CPW width 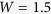 *μ*m are kept constant. In calculations, we take 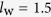 *μ*m and 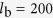 nm. The mf magnetic field distribution below the CPW depends now also on the *x* coordinate, i.e., 
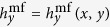
. To match the mesh cells of MSs, the field profiles used in MSs was constructed by interpolating field profiles 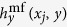 obtained from CST simulations at several values of *x*, 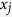, by using the following linear homotopic transformation providing a linear variation of the line width between points 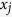 and 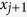:


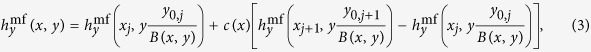


where 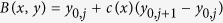 and 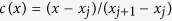. The symbol 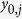 denotes the zero of 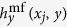 on the half-line *y* > 0. This approach was introduced to conserve the proper position of the zeros of the field in the transition region in order to provide a smoother, higher quality interpolation of the field.The sampling points were spaced 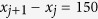 nm apart. The map of the *y* component of the mf magnetic field generated by this nonuniform CPW at the top surface of the ferromagnetic film is shown in Fig. 4c. Since the YIG film is very thin (10 nm), the magnetic field is almost uniform across its whole thickness. Figure 4d shows the distribution of the dynamic magnetic field 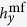 in the YIG film along the *x* axis, i.e., the symmetry axis of the signal line.

The dynamic component of the magnetization vector of an SW generated at 22.12 GHz with the aid of the proposed CPW in the homogeneous YIG film is plotted in Fig. 4b. The beam-type behavior of the SW is evident, so that the basic idea of the suggested approach is justified. As expected, the beam remains well convergent even at large distances from the transducer. The SW beam width at the half-power level is 1.5 *μ*m in the beam waist lying at *y* = 0. This width can be controlled by adjusting the length of the transducer section having the profile of CPW_1_, 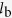, and the size and curvature of the transition section, 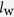, as will be shown in the next paragraph. The minimum achievable beam width is likely to be restricted by the available nanofabrication technology.

### Advanced regimes

Since the basic mechanism of SW beam generation in YIG film has now been numerically validated, it is worthwhile to consider further design possibilities and novel excitation scenarios.

Preliminary design of a nonuniform CPW made up of sections creating a mf magnetic field whose FT is either large or small at a required wave number can be done using a simple analytical model. Provided that the signal and ground lines in a CPW are wide enough, the current density is concentrated near the edges of the lines adjacent to the gaps, due to skin and proximity effects^31^. In the first approximation, this current distribution can be modeled by a system of four infinitesimally thin wires located at the edges of the gaps, with the current density given by


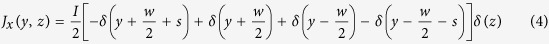


where *I* is the current flowing through the signal line and *δ*(·) is the Dirac delta. The slow oscillatory dependence on the *x* coordinate is neglected. Biot-Savart’s law yields the magnetic field generated by this current distribution; the FT of its *y* component is


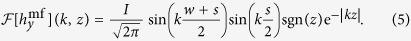


It follows that the first maximum and the first zero of the FT occur at


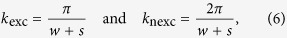


respectively. Notably, their positions depend solely on the distance between the centers of the two gaps, 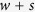. Therefore, a CPW with 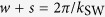 containing a short section with 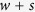 reduced to 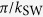 should be a good beam generator for SWs with wave number 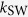. The distances between the center of the gaps in CPW_1_ and CPW_2_ are 160 and 355 nm, respectively. Thus, they are quite close to the values of 175 and 350 nm obtained from the analytical model for 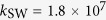 m^−1^.

**Figure 5:**
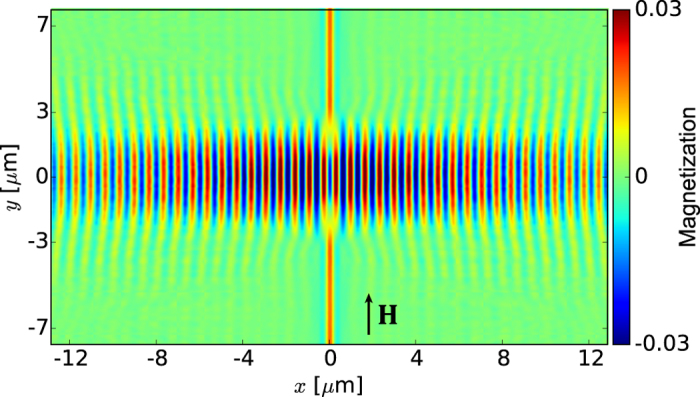
Excitation of a SW beam in the Damon-Eshbach geometry in a thin YIG film. The film is saturated by an in-plane static external magnetic field of magnitude 0.5 T. The SW beam propagates perpendicularly to the direction of the magnetic field (results of MSs).

The proposed approach to SW beam generation is very simple and practical. Although the numerical demonstration has initially been done for the dielectric film magnetized normally to the film plane, the same mechanism works for other geometries and different ferromagnetic films, whenever the mf magnetic field generated by a CPW can induce magnetization precession below the transducer. This includes the Damon-Eshbach geometry (i.e., a thin ferromagnetic film with in-plane magnetization and SW propagation perpendicular to the magnetization) and ferromagnetic metallic films at low- or high-frequency parts of the SW spectra, with anisotropic and almost isotropic dispersion relation, respectively. However, in the latter case the influence of the metal’s properties on the 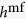 field generated by the CPW needs to be taken into account. An example SW beam generated in the Damon-Eshbach geometry in a YIG film is shown in Fig. 5. In this case, the dimensions of the nonuniform CPW from Fig. 4 are scaled by factor of 2 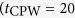 nm, 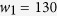 nm, 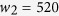 nm, 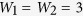 *μ*m, 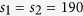 nm), while the external magnetic field of magnitude 0.5 T is in-plane and directed along the CPW axis. The excitation frequency is 17.29 GHz.

**Figure 6:**
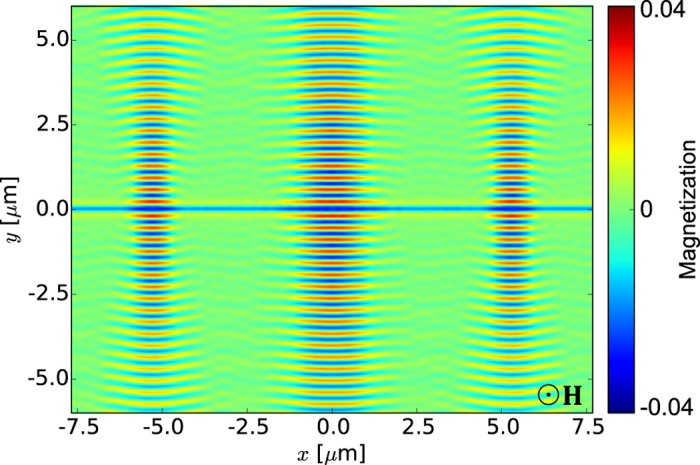
Excitation of three parallel in-phase, SW beams of different widths with a single nonuniform CPW. Dynamic component of the magnetization vector for three SW beams formed simultaneously in a thin YIG film by the mf magnetic field generated by a single CPW of a complex shape (results of MSs). The left and right beams, generated by CPW sections with 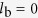 nm and 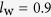 *μ*m, are narrower than the middle beam, generated by a CPW section with 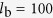 nm and 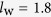 *μ*m. The YIG film of thickness 20 nm is saturated along the normal to the film plane by an external magnetic field of magnitude 1 T.

Multiple modulated sections can also be introduced into a CPW to generate multiple coherent SW beams in a homogeneous ferromagnetic film. An example is presented in Fig. 6. As the beam divergence is weak, individual beams do not interfere with each other in the considered space region. Each beam may have different characteristics, such as width and intensity, since they are determined by the geometry of the local CPW nonuniformity. This opens up a route to multichannel structures for SWs created simply by modulating the microwave magnetic field through local modifications of the transducer geometry, with no need to make either the ferromagnetic film inhomogeneous or the biasing magnetic field nonuniform.

## Discussion

The possibilities discussed above do not exhaust the broad range of potential applications of SW beam magnonics. For instance, demultiplexer-type operation might be achieved by creating such a distribution of 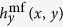 that injection of a mixed dual-frequency microwave signal into the CPW would cause efficient generation of SW beams at two distinct frequencies within different regions of the YIG film, associated with different virtual propagation channels. However, to realize this regime, additional design efforts are required, e.g., to limit excitation at undesirable frequencies in both channels. SW beams can also be utilized for sensing, transmitting information and performing logic operations. In particular, the concepts of logic elements based on interference effects, such as Mach-Zehnder interferometers, can also be adapted for SW beam based operation, provided that a mechanism for introducing a controllable phase shift between two SW beams is available. In this case, reading can be done by direct beam interference^32^ (this will require a change of the beam propagation direction) or using a detecting CPW transducer of appropriate width^33^,^34^.

To summarize, we have proposed to use nonuniform microwave CPW transducers for efficient local excitation of SW beams in thin ferromagnetic films. Modulation of the transducer geometry ensures that the necessary condition of SW generation, the match between the SW wave vector and the wave vector corresponding to the maximum of the spatial Fourier transform of the microwave magnetic field induced by the transducer, is satisfied only locally, i.e., in a short section of the waveguide. The beam characteristics, such as waist width, are controlled by the geometry of the modulated transducer section. The presented numerical examples demonstrate the broad potential of the proposed approach, which holds promise as a platform for future SW based circuitry. In particular, we have shown that SW beams can be generated in different magnetization configurations and that multiple SW beams associated with separate virtual propagation channels can be generated in one thin homogeneous ferromagnetic film. Manipulation of SW beams in thin films and their application to graded-index magnonics^35^, in particular in magnonic devices, will be the subject of upcoming research.

## Methods

### Micromagnetic simulations

The results of MSs reported in this paper have been obtained with the open-source software MuMax3 employing the finite difference method^36^. We used a finite difference grid with resolution 5 nm in the *x* and *y* directions (i.e., in the film plane) and 20 nm in the *z* direction (along the normal to the film surface), and the Landau-Lifshitz equation was solved using the RK45 (Dormand-Prince) method^37^.

The MSs were performed for a uniformly magnetized thin YIG film. The simulated structures were large enough to prevent undesired boundary effects from having a significant influence on SW dynamics in the central part of the film. Moreover, absorbing boundaries were introduced at the edges of the computational domain parallel to the transducer axis (i.e., at 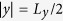, where 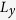 is the size of the computational domain in the *y* direction); see the supplementary material to ref. 38. In addition, periodic boundary conditions were applied on the edges perpendicular to the transducer axis (i.e., at 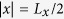, where 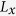 is the size of the computational domain in the *x* direction) to avoid the effects of abrupt symmetry breaking of the dynamic magnetic field.

In MSs, in order to visualize the low divergence of excited beams far from the excitation point, a low damping parameter 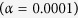, one order of magnitude smaller than for YIG films, has been assumed. However, increasing the damping does not interfere with the generation of SW beams but only limits their propagation distance.

### Electromagnetic simulations

The distributions of the magnetic fields generated by CPWs and used to excite the SWs have been calculated with CST Microwave Studio, a commercial Maxwell solver based on the finite integration method. The magnitude of the mf magnetic field used to excite SWs was sufficiently small to preserve linear magnetization dynamics induced in ferromagnetic films.

## Additional Information

**How to cite this article**: Gruszecki, P. *et al.* Microwave excitation of spin wave beams in thin ferromagnetic films. *Sci. Rep.* **6**, 22367; doi: 10.1038/srep22367 (2016).
